# Author Correction: Comprehensive mutational scanning of EGFR reveals TKI sensitivities of extracellular domain mutants

**DOI:** 10.1038/s41467-024-47675-w

**Published:** 2024-04-16

**Authors:** Tikvah K. Hayes, Elisa Aquilanti, Nicole S. Persky, Xiaoping Yang, Erica E. Kim, Lisa Brenan, Amy B. Goodale, Douglas Alan, Ted Sharpe, Robert E. Shue, Lindsay Westlake, Lior Golomb, Brianna R. Silverman, Myshal D. Morris, Ty Running Fisher, Eden Beyene, Yvonne Y. Li, Andrew D. Cherniack, Federica Piccioni, J. Kevin Hicks, Andrew S. Chi, Daniel P. Cahill, Jorg Dietrich, Tracy T. Batchelor, David E. Root, Cory M. Johannessen, Matthew Meyerson

**Affiliations:** 1 Department of Medical Oncology, Dana-Farber Cancer Institute & Harvard Medical School, Boston, MA USA; 2 Cancer Program, The Broad Institute of M.I.T. and Harvard, Cambridge, MA USA; 3 Genetic Perturbation Platform, The Broad Institute of M.I.T. and Harvard, Cambridge, MA USA; 4 Data Science Platform, The Broad Institute of M.I.T. and Harvard Cambridge, Cambridge, MA USA; 5 Summer Honors Undergraduate Research Program, Harvard Medical School, Boston, MA USA; 6 Department of Pathology, H. Lee Moffitt Cancer Center and Research Institute, Tampa, FL USA; 7 Center for Neuro-Oncology, Division of Neuro-Oncology, Massachusetts General Hospital, Boston, MA USA; 8 Department of Neurology, Division of Neuro-Oncology, Massachusetts General Hospital, Boston, MA USA; 9 Department of Neurology, Brigham and Women’s Hospital & Harvard Medical School, Boston, MA USA; 10 Present Address: Department of Molecular and Medical Pharmacology, University of California, Los Angeles, CA USA; 11 Present Address: Aera Therapeutics, Cambridge, MA USA; 12 Present Address: Merck Research Laboratories, Cambridge, MA USA; 13 Present Address: Department of Oncology, Novartis Institutes for Biomedical Research, Cambridge, MA USA

**Keywords:** Cancer genomics, CNS cancer, Non-small-cell lung cancer

Correction to: *Nature Communications* 10.1038/s41467-024-45594-4, published online 28 March 2024

The original version of this Article contained an error in Fig. 4b, in which the symbols for LacZ and EGFR WT were switched. This has been corrected in both the PDF and HTML versions of the Article.

