# Supplementary material for: Comprehensive mutational scanning of EGFR reveals TKI sensitivities of extracellular domain mutants
Source: Nat Commun. 2024 Mar 28;15:2742. doi: 10.1038/s41467-024-45594-4 (PMC10978866; doi:10.1038/s41467-024-45594-4)
Supplement: Supplementary file 1 — Supplementary Information [file 41467_2024_45594_MOESM1_ESM.pdf]

Supplementary Figure 1

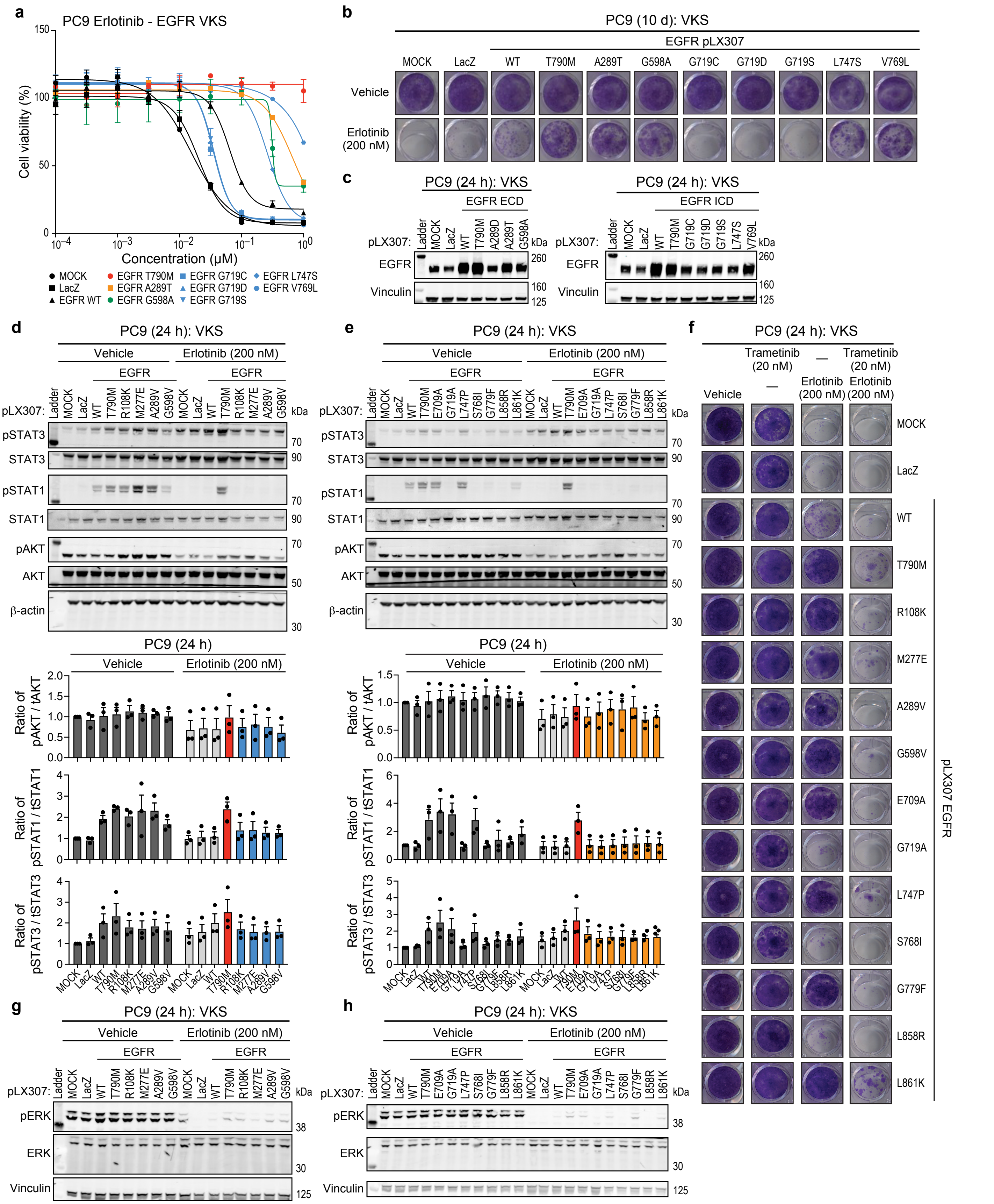

Supplementary Fig. 1

Characterization of additional variants of known significance (VKS) in the PC9 oncogene addiction model. a, PC9 cell lines expressing either LacZ, EGFR WT, EGFR T790M, EGFR extracellular domain variants (A289T or G598A), or EGFR intracellular domain variants (G719C, G719D, G719S, L747S, or V769L), after 144 h treatment with increasing doses of erlotinib (normalized to vehicle control). A representative experiment is shown, remaining biological replicates are located in Source Data (N=3). Data are presented as mean values +/- SD. **b,** Colony formation assay in PC9 EGFR mutants and controls as in **(a)** after a 10 d of treatment with either vehicle (DMSO) or 200 nM erlotinib. A representative experiment is shown (N=3). **c,** Representative immunoblots displaying the effects of PC9 EGFR mutants and controls as in **(a)** on expression levels of total EGFR. Vinculin is the loading control (N=3). **d-e,** Representative immunoblots displaying the effect of 200 nM erlotinib after 24 h treatment on PC9 cell lines expressing either LacZ, EGFR WT, EGFR T790M, EGFR extracellular domain variants (R108K, M277E, A289V, or G598V) **(d)**, or EGFR intracellular domain variants (E709A, G719A, L747P, S768I, G779F, L858R or L861K) **(e)** on the levels of phosphorylated STAT1, STAT3, and AKT and total STAT1, STAT3, and AKT. β-actin is the loading control. Normalization and quantification of phospho- AKT, -STAT1, and -STAT3 to total AKT, STAT1, and STAT3 protein levels. Data are presented as mean values +/- SEM of biological replicates (N=3). **f,** Colony formation assay in PC9 EGFR mutants and controls as in **(d)** and **(e)** after a 10 d of treatment with either vehicle (DMSO), 20 nM trametinib, 200 nM erlotinib, or 20 nM trametinib/200 nM erlotinib. A representative experiment is shown (N=3). **g-h,** Representative immunoblots displaying the effect of 20 nM trametinib/200 nM erlotinib after 24 h treatment on PC9 cell lines expressing either LacZ, EGFR WT, EGFR T790M, EGFR extracellular domain variants (R108K, M277E, A289V, or G598V) **(g)**, or EGFR intracellular domain variants (E709A, G719A, L747P, S768I, G779F, L858R or L861K) **(h)** on the levels of phosphorylated and total ERK. Vinculin immunoblotting was used to determine equivalent loading (N=3).

Supplementary Figure 2

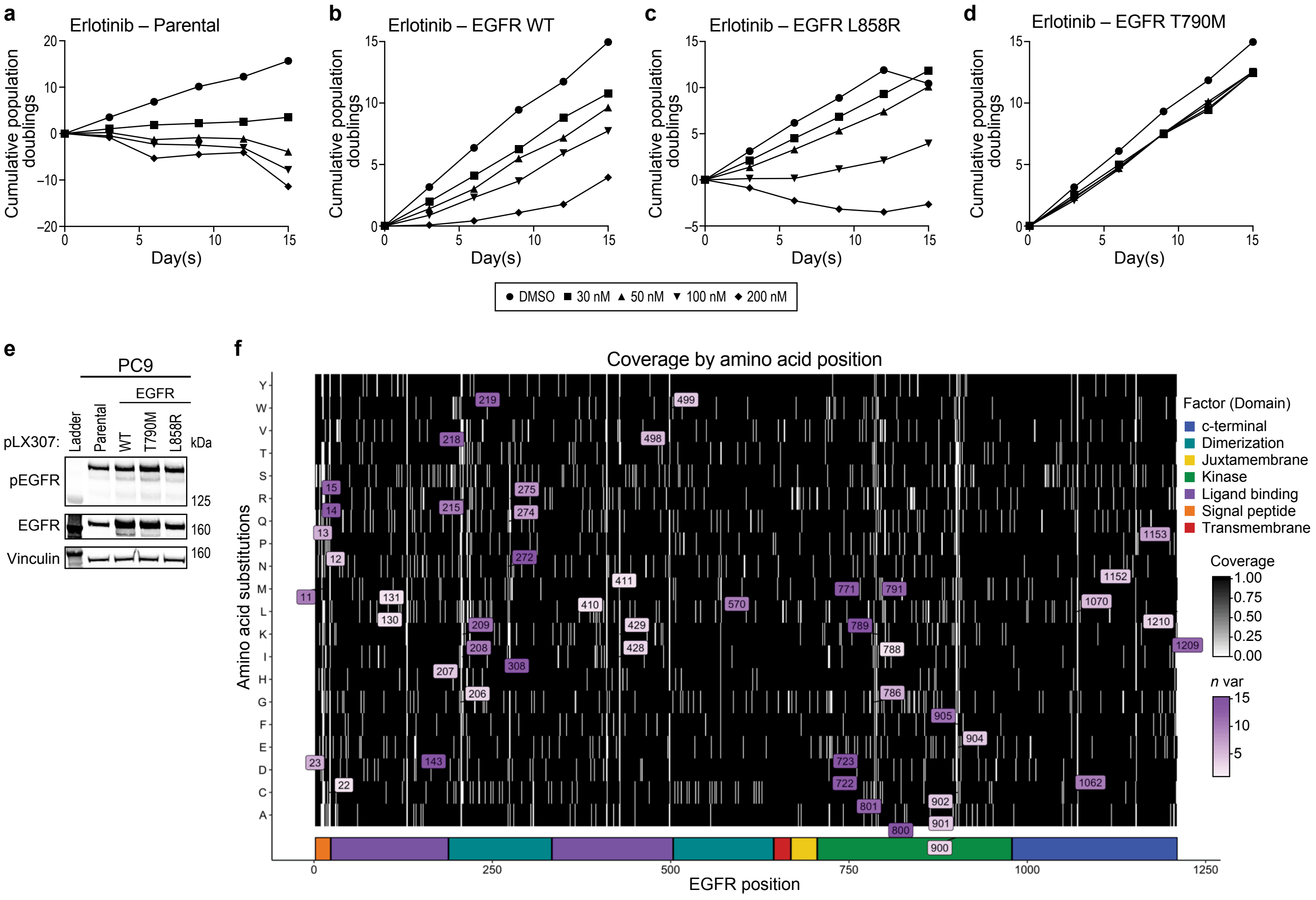

Supplementary Fig. 2

Deep Mutational Scanning Screen Optimization. a-d, Cumulative population doublings for PC9 cell lines expressing screening controls, parental **(a)**, EGFR WT **(b)**, EGFR L858R **(c)** or EGFR T790M **(d)** after 15 d treatment with either DMSO, 30 nM, 50 nM, 100 nM, or 200 nM of erlotinib. Data are presented as mean values of technical replicates (N=1) **e,** Representative immunoblots displaying the effects of PC9 cell lines expressing either EGFR WT, EGFR T790M, or EGFR L858R on the levels of total phosphorylated and total EGFR. Vinculin immunoblotting was used to determine equivalent loading. **f,** Deep mutational screen analysis amino acid coverage.

Supplementary Figure 3

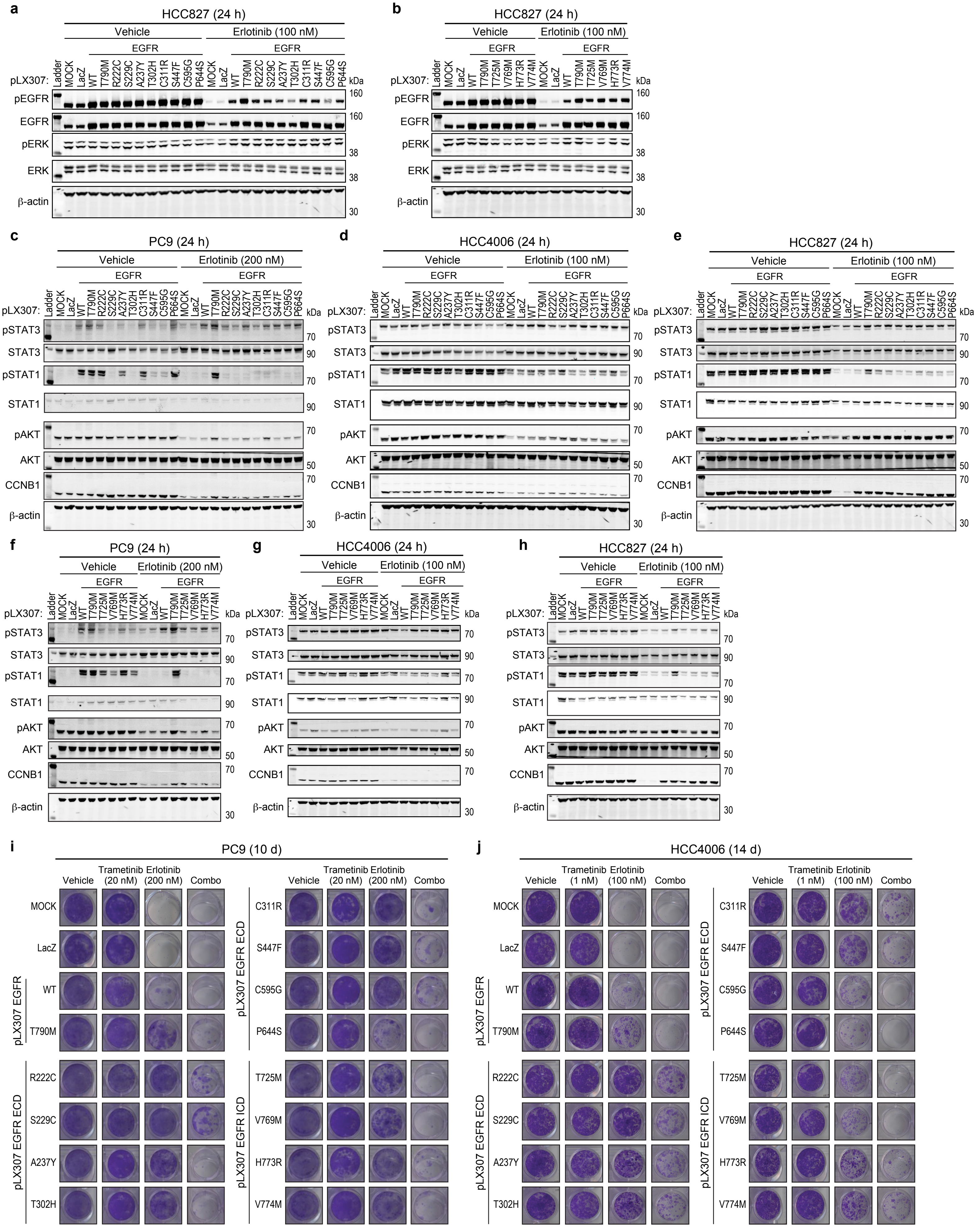

Supplementary Fig. 3

Additional characterization of EGFR variants of unknown significance (VUS) in lung cancer models. **a-b,** Representative immunoblots displaying the effect of 100 nM erlotinib after 24 h treatment of HCC827 cell lines expressing either LacZ, EGFR WT, EGFR T790M, EGFR extracellular domain variants (R222C, S229C, A237Y, T302H, C311R, S447F, C595G, or P644S) **(a)**, or EGFR intracellular domain variants (T725M, V769M, H773R, and V774M) **(b)** on the levels of both phosphorylated EGFR and ERK and total EGFR and ERK. β-actin immunoblotting was used to determine equivalent loading (N=3). **c-e,** Representative immunoblots displaying the effect of either 200 nM (PC9) **(c)**, 100 nM (HCC4006) **(d)**, or 100 nM (HCC827) **(e)** erlotinib after 24 h treatment of cell lines expressing EGFR mutants and controls as in **(a)** on the levels of phosphorylated STAT1, STAT3, and AKT and total STAT1, STAT3, AKT, Cyclin B1. β-actin immunoblotting was used to determine equivalent loading (N=3). **f-h,** Representative immunoblots displaying the effect of either 200 nM (PC9) **(f)**, 100 nM (HCC4006) **(g)**, or 100 nM (HCC827) **(h)** erlotinib after 24 h treatment of cell lines expressing EGFR mutants and controls as in **(b)** on the levels of phosphorylated STAT1, STAT3, and AKT and total STAT1, STAT3, AKT, Cyclin B1. β-actin immunoblotting was used to determine equivalent loading (N=3). **i,** Colony formation assay in PC9 expressing EGFR mutants and controls as in **(a,b)** after a 10 d of treatment with either vehicle (DMSO), 20 nM trametinib, 200 nM erlotinib, or 20 nM trametinib/200 nM erlotinib. A representative experiment is shown (N=3). **j,** Colony formation assay in HCC4006 cell lines expressing EGFR mutants and controls as in **(a,b)** after a 14 d of treatment with either vehicle (DMSO), 1 nM trametinib, 100 nM erlotinib, or 1 nM trametinib/100 nM erlotinib. A representative experiment is shown (N=3).

Supplementary Figure 4

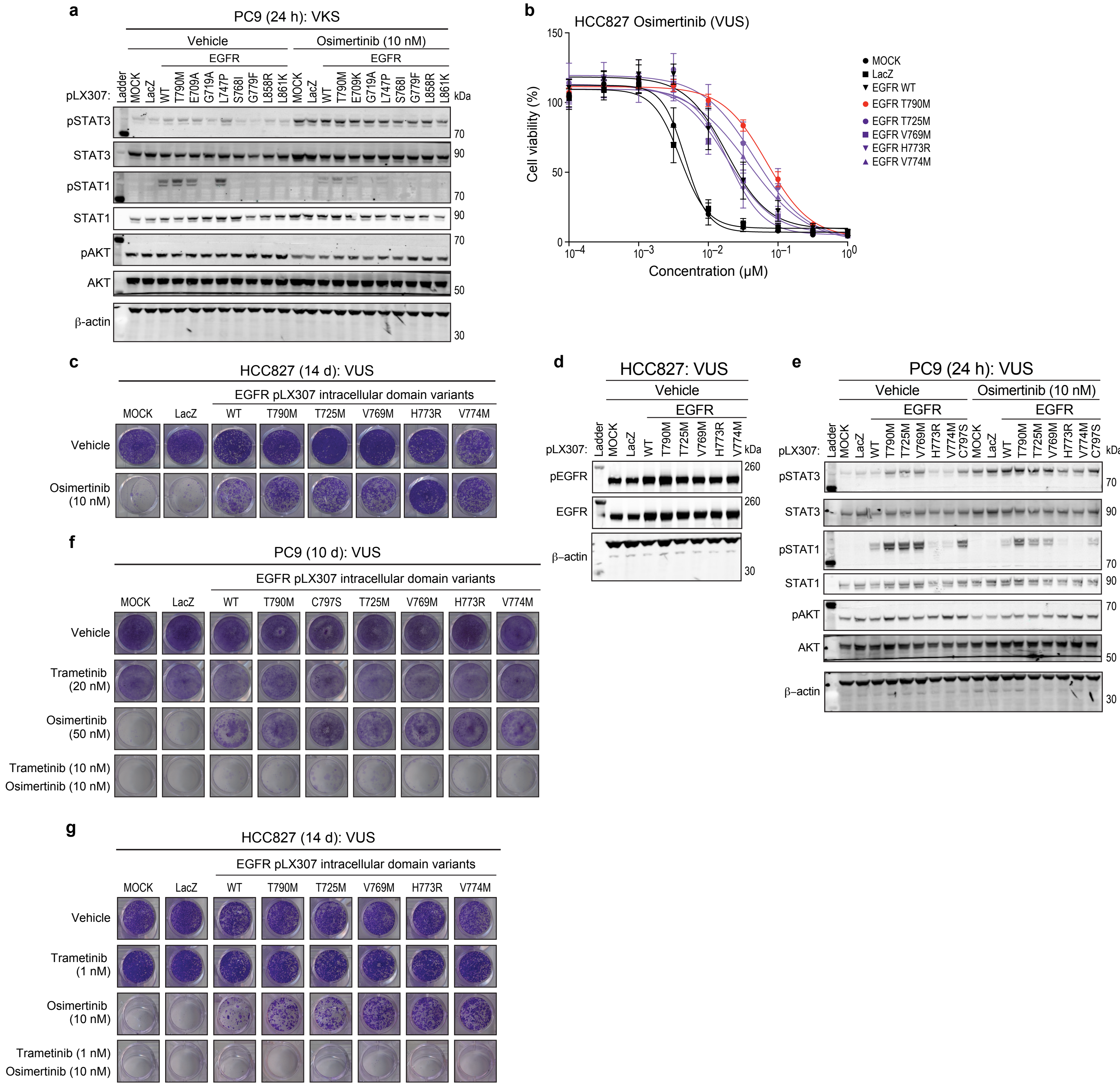

Supplementary Fig. 4

Additional effects of osimertinib treatment in lung cancer models with intracellular domain variants. a, Representative immunoblots displaying the effect of 10 nM osimertinib after 24 h treatment on PC9 cell lines expressing either LacZ, EGFR WT, EGFR T790M, or EGFR VKS intracellular domain variants (E709A, G719A, L747P, S768I, G779F, L858R or L861K) on the levels of phosphorylated STAT1, STAT3, and AKT and total STAT1, STAT3, and AKT. β-actin immunoblotting was used to determine equivalent loading (N=3). **b,** HCC827 cell lines expressing either LacZ, EGFR WT, EGFR T790M, or EGFR VUS intracellular domain variants (T725M, V769M, H773R, and V774M) after 144 h treatment with increasing doses of osimertinib (normalized to vehicle control). A representative experiment is shown, remaining biological replicates are located in Source Data (N=3). Data are presented as mean values +/- SD. **c,** Colony formation with HCC827 expressing EGFR mutants and controls as in **(b)** after 14 d of 10 nM osimertinib treatment. A representative experiment is shown (N=3). **d,** Representative immunoblots displaying the effects of HCC827 cell lines expressing EGFR mutants and controls as in **(b)** expression levels of phosphorylated and total EGFR. β-actin immunoblotting was used to determine equivalent loading (N=3). **e,** Representative immunoblots displaying the effect of 10 nM osimertinib after 24 h treatment on PC9 cell lines expressing EGFR mutants and controls as in **(b)** on the levels of phosphorylated STAT1, STAT3, and AKT, and total STAT1, STAT3, and AKT. β-actin immunoblotting was used to determine equivalent loading (N=3). **f-g,** Colony formation assay in PC9 **(f)** or HCC827 **(g)** cell lines expressing EGFR mutants and controls as in **(b)** after a 14 d of treatment with either vehicle (DMSO), 20 nM (PC9) or 1 nM (HCC827) trametinib, 50 (PC9) or 1 (HCC827) nM osimertinib, or 10 nM trametinib/10 nM osimertinib (PC9) or 1 nM trametinib/10 nM osimertinib (HCC827). A representative experiment is shown (N=3).

Supplementary Figure 5

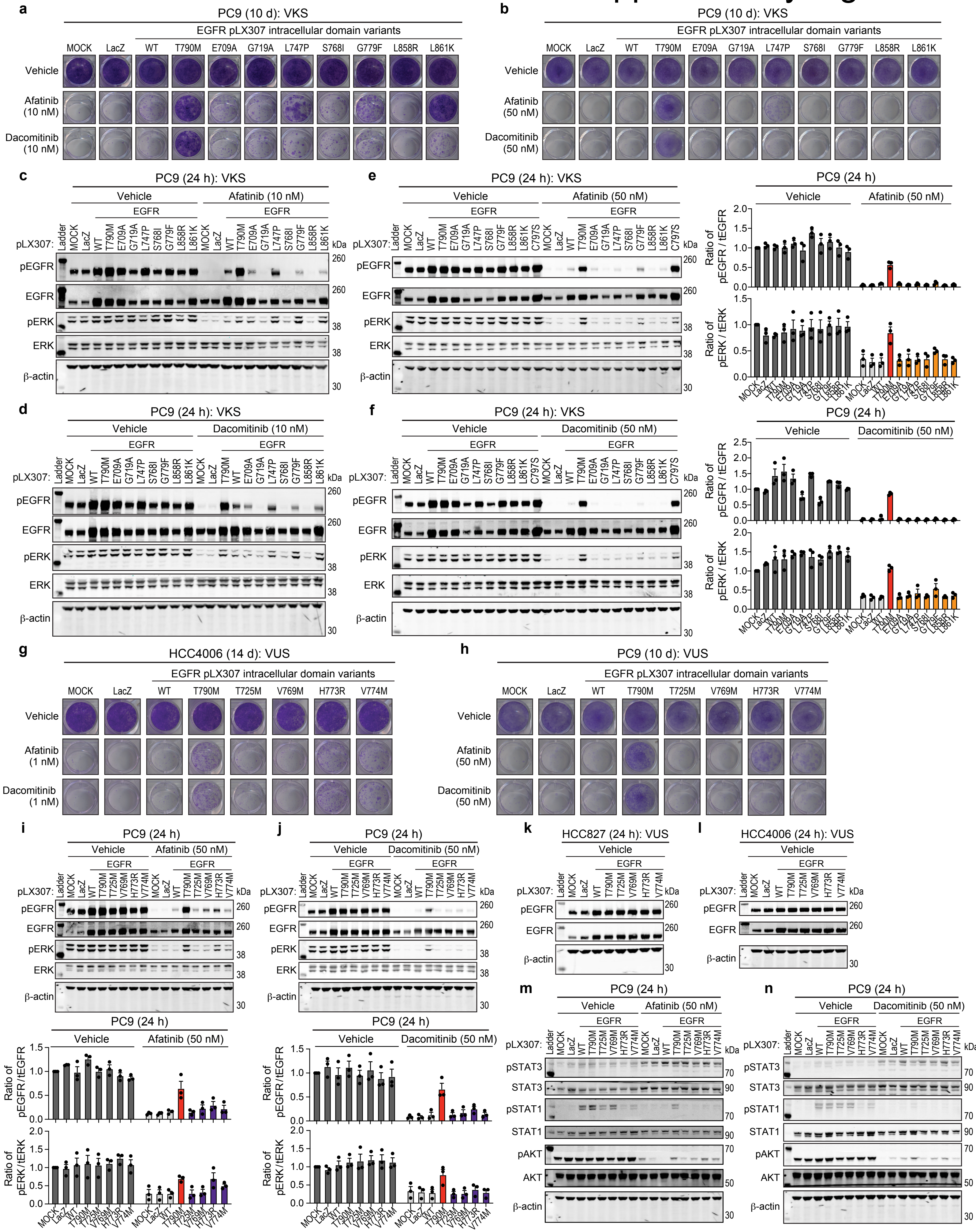

Supplementary Fig. 5

Additional effects of afatinib and dacomitinib treatment in lung cancer models with intracellular domain variants. a-b, Colony formation with 10 nM **(a)** or 50 nM **(b)** afatinib or dacomitinib after 10 d treatment on PC9 cell lines expressing controls or EGFR VKS intracellular domain variants. A representative experiment is shown (N=3). **c-d,** PC9 cell lines expressing EGFR mutants and controls on expression levels of phosphorylated EGFR and ERK and total EGFR and ERK after 24 h treatment with 10 nM afatinib **(c)** or dacomitinib **(d)**. β-actin is the loading control (N=3). **e-f,** PC9 cell lines expressing EGFR mutants and controls on expression levels of phosphorylated EGFR and ERK and total EGFR and ERK after 24 h treatment with 50 nM afatinib **(e)** or dacomitinib **(f)**. β-actin is the loading control. Normalization and quantification of phospho-EGFR and -ERK to total-EGFR and -ERK protein levels. Presented data are mean values +/-SEM of biological replicates (N=3). **g-h,** Colony formation with HCC4006 **(g)** or PC9 **(h)** cell lines expressing either controls or EGFR VKS intracellular domain variants. Cells were treated with either 1 nM of afatinib or dacomitinib (HCC4006) or 50 nM of afatinib or dacomitinib (PC9) for 24 h. A representative experiment is shown (N=3). **i-j,** Representative immunoblots displaying the effects of PC9 cell lines expressing EGFR mutants and controls on expression of phosphorylated EGFR and ERK and total EGFR and ERK after 24 h treatment with either afatinib **(i)** or dacomitinib **(j)**. β-actin is the loading control. Normalization and quantification of phospho-EGFR and -ERK to total-EGFR and -ERK protein levels. Presented data are mean values +/-SEM of biological replicates (N=3). **k-l,** Representative immunoblots displaying the effects of HCC827 **(k)** or HCC4006 **(l)** cell lines expressing EGFR mutants and controls on expression levels of phosphorylated and total EGFR. β-actin is the loading control (N=3). **m-n,** Representative immunoblots displaying the effect of 50 nM afatinib or dacomitinib after 24 h treatment on PC9 cell lines expressing EGFR mutants and controls on the levels of phosphorylated STAT1, STAT3, and AKT, and total STAT1, STAT3, and AKT. β-actin is the loading control (N=3).

Supplementary Figure 6

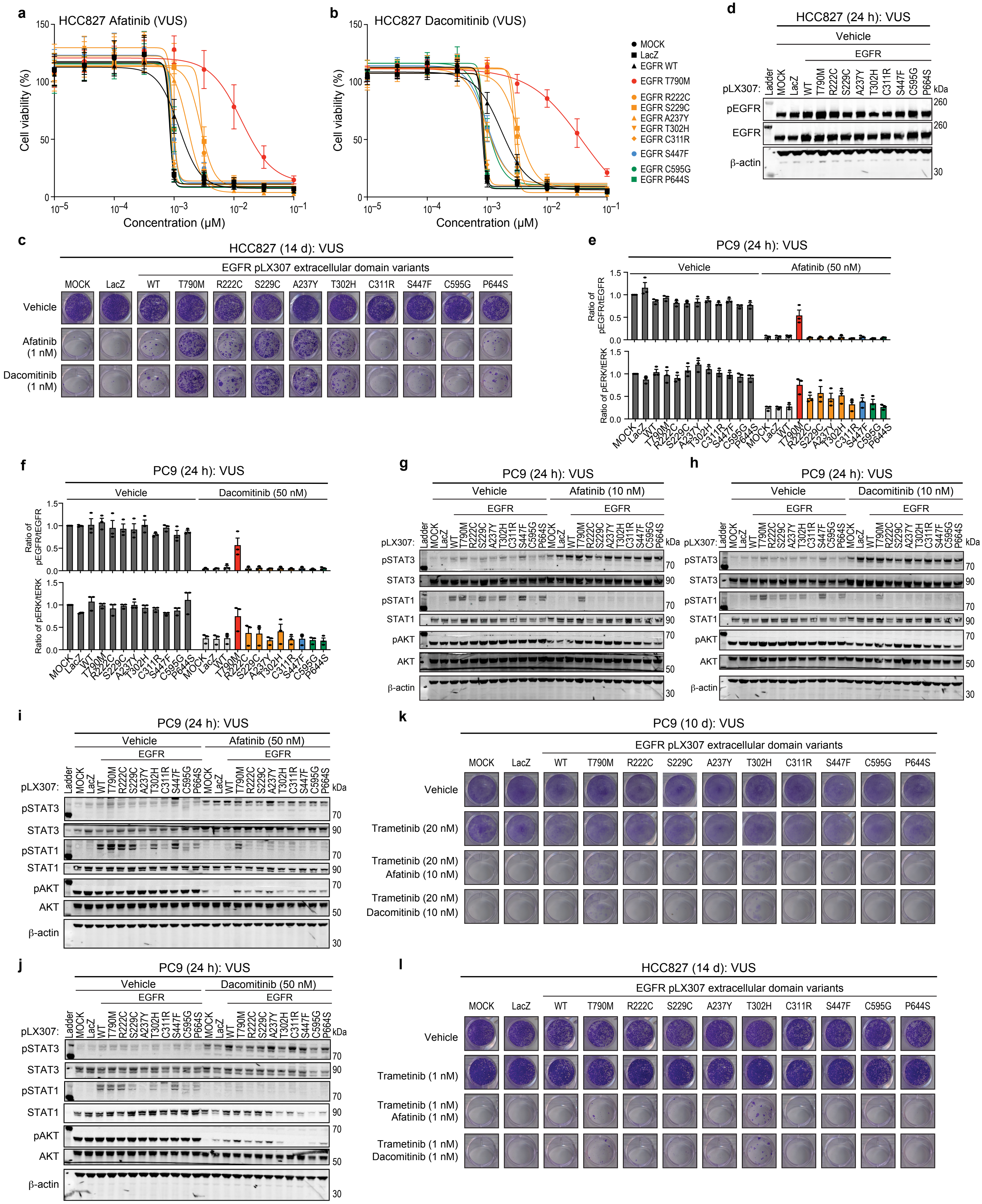

Supplementary Fig. 6

Additional effects of afatinib and dacomitinib treatment in lung cancer models with extracellular domain variants. a-b, HCC827 cell lines expressing either controls or EGFR extracellular domain variants after 144 h treatment with increasing doses of afatinib **(a)** or dacomitinib **(b)**. A representative experiment is shown, remaining biological replicates are located in Source Data (N=3). Data are presented as mean values +/- SD. **c,** Colony formation with HCC827 cell lines expressing EGFR mutants and controls as in **(a)** after 14 d of treatment with either vehicle (DMSO), 1 nM afatinib or 1 nM dacomitinib. A representative experiment is shown (N=3). **d,** Representative immunoblots displaying the effects of HCC827 cell lines expressing EGFR mutants and controls on expression levels of phosphorylated and total EGFR. β- actin is the loading control. **e-f,** Normalization and quantification of phospho-EGFR and - ERK to total-EGFR and -ERK protein levels after 24 h treatment with either 50 nM of afatinib **(e)** or dacomitinib **(f)** in PC9 cell lines expressing EGFR mutants and controls. Data are presented as mean values +/- SEM of biological replicates (N=3). **g-h,** Representative immunoblots displaying the effect of either 10 nM of afatinib **(g)** or dacomitinib **(h)** after 24 h treatment on PC9 cell lines expressing EGFR mutants and controls on the levels of phosphorylated STAT1, STAT3, and AKT, and total STAT1, STAT3, and AKT. β-actin immunoblotting was used to determine equivalent loading (N=3). **i-j,** Representative immunoblots displaying the effect of either 50 nM of afatinib **(i)** or dacomitinib **(j)** after 24 h treatment on PC9 cell lines expressing EGFR mutants and controls as in **(a,b)** on the levels of phosphorylated STAT1, STAT3, and AKT, and total STAT1, STAT3, and AKT. β-actin is the loading control (N=3). **k-l,** Colony formation assay in PC9 **(k)** and HCC827 **(l)** cell lines expressing EGFR mutants and controls after a 10-14 d of treatment with either vehicle (DMSO), 1 nM or 20 nM trametinib, or 1 nM trametinib/1 nM afatinib or 20 nM trametinib/10 nM afatinib, or 1 nM trametinib/1 nM dacomitinib or 20 nM trametinib/10 nM dacomitinib. A representative experiment is shown (N=3).

Supplementary Figure 7

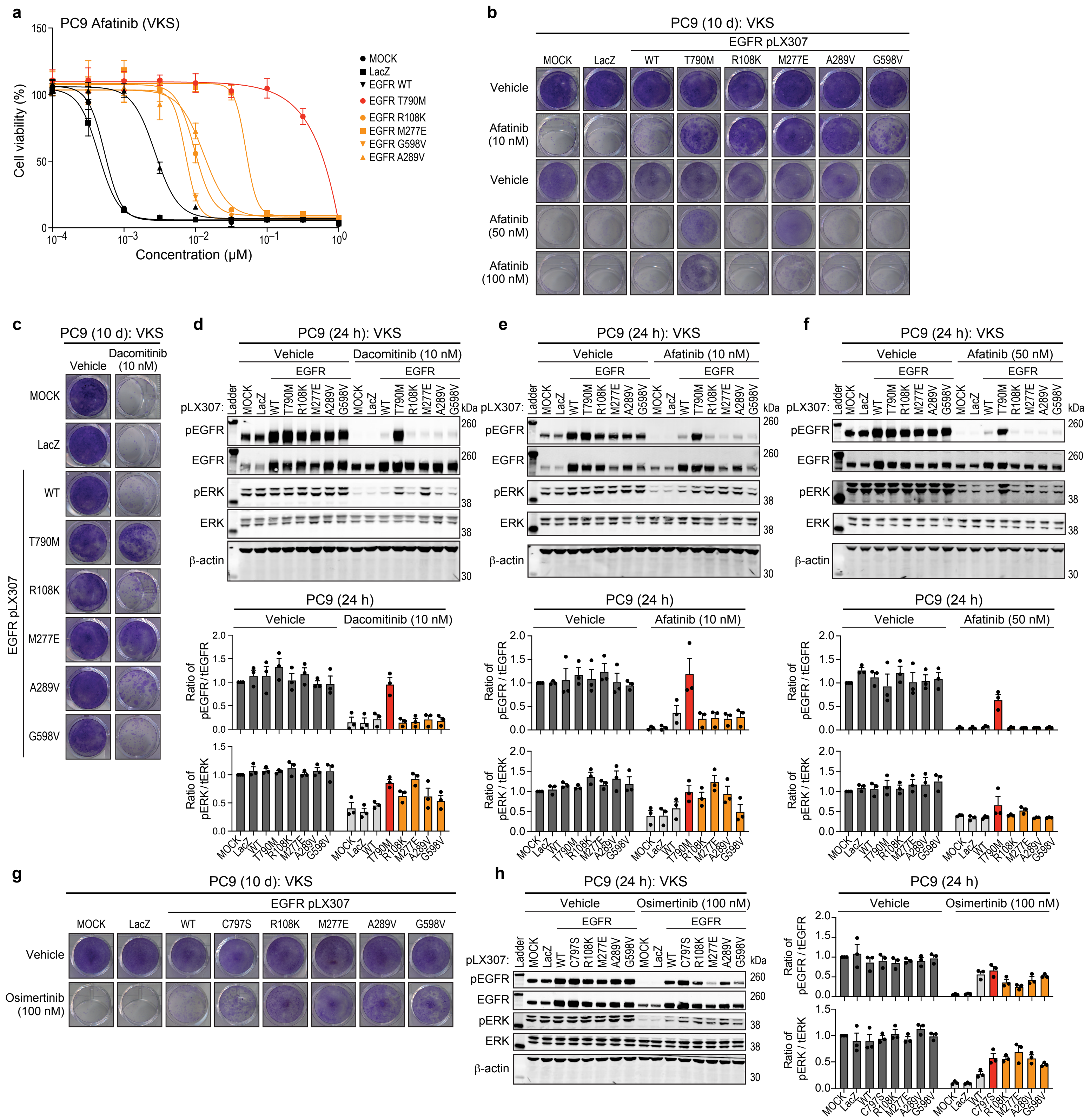

Supplementary Fig. 7

Additional effects of second-generation inhibitors on EGFR extracellular domain VKS. a, PC9 cell lines expressing either LacZ, EGFR WT, EGFR T790M, EGFR VKS extracellular domain variants (R108K, M277E, A289V, or G598V) after 144 h treatment with increasing doses of afatinib. A representative experiment is shown, remaining biological replicates are located in Source Data (N=3). Data are presented as mean values +/- SD. **b,** Colony formation with PC9 cell lines expressing EGFR mutants and controls as in **(a)** after 10 d of treatment with either vehicle (DMSO), 10, 50, or 100 nM afatinib. A representative experiment is shown (N=3). **c,** Colony formation with PC9 cell lines expressing EGFR mutants and controls after 10 d of treatment with either vehicle (DMSO), 10 nM dacomitinib. A representative experiment is shown (N=3). **d-f,** Representative immunoblots displaying the effect of 10 nM dacomitinib **(d)** and 10 nM **(e)** or 50 nM **(f)** of afatinib after 24 h treatment on PC9 cell lines expressing EGFR mutants and controls on the levels of both phosphorylated EGFR and ERK and total EGFR and ERK. β-actin is the loading control. Normalization and quantification of phospho-EGFR and -ERK to total-EGFR and -ERK protein levels **(below)**. Data are presented as mean values+/- SEM of biological replicates (N=3). **g,** Colony formation with PC9 cell lines expressing EGFR mutants and controls after 10 d of treatment with either vehicle (DMSO), 100 nM osimertinib. A representative experiment is shown (N=3) **h,** Representative immunoblots displaying the effect of 100 nM osimertinib after 24 h treatment on PC9 cell lines expressing EGFR mutants and controls on the levels of both phosphorylated EGFR and ERK and total EGFR and ERK. β-actin immunoblotting was used to determine equivalent loading. Normalization and quantification of phospho-EGFR and -ERK to total-EGFR and -ERK protein levels (**right**). Data are presented as mean values +/- SEM of biological replicates (N=3).

Supplementary Table 1

z-score	Total number of functional variants identified from screen	Total number of functional variant overlap from previous studies	Total number of new functional variants identified in the screen	Percent new functional variants (%)
> 4	88	4	84	95.5
> 3	216	9	207	95.8
> 2	559	20	539	96.4

Supplementary Table 1. Identification of novel EGFR variants. Table describing the percentage of novel EGFR variants observed in the screen based on Z-score enrichment.
