# Supplementary material for: Comprehensive mutational scanning of EGFR reveals TKI sensitivities of extracellular domain mutants
Source: Nat Commun. 2024 Mar 28;15:2742. doi: 10.1038/s41467-024-45594-4 (PMC10978866; doi:10.1038/s41467-024-45594-4)
Supplement: Supplementary file 2 — Reporting Summary [file 41467_2024_45594_MOESM2_ESM.pdf]

nature portfolio

Corresponding author(s): Matthew Meyerson

Last updated by author(s): 10/25/2023

Reporting Summary

Nature Portfolio wishes to improve the reproducibility of the work that we publish. This form provides structure for consistency and transparency in reporting. For further information on Nature Portfolio policies, see our Editorial Policies and the Editorial Policy Checklist.

Statistics

For all statistical analyses, confirm that the following items are present in the figure legend, table legend, main text, or Methods section.

n/a	Confirmed	
x		The exact sample size (*n*) for each experimental group/condition, given as a discrete number and unit of measurement
	x	A statement on whether measurements were taken from distinct samples or whether the same sample was measured repeatedly
	x	The statistical test(s) used AND whether they are one- or two-sided<br>*Only common tests should be described solely by name; describe more complex techniques in the Methods section.*
x		A description of all covariates tested
	x	A description of any assumptions or corrections, such as tests of normality and adjustment for multiple comparisons
x		A full description of the statistical parameters including central tendency (e.g. means) or other basic estimates (e.g. regression coefficient) AND variation (e.g. standard deviation) or associated estimates of uncertainty (e.g. confidence intervals)
	x	For null hypothesis testing, the test statistic (e.g. *F*, *t*, *r*) with confidence intervals, effect sizes, degrees of freedom and *P* value noted<br>*Give P values as exact values whenever suitable.*
x		For Bayesian analysis, information on the choice of priors and Markov chain Monte Carlo settings
x		For hierarchical and complex designs, identification of the appropriate level for tests and full reporting of outcomes
x		Estimates of effect sizes (e.g. Cohen's *d*, Pearson's *r*), indicating how they were calculated

Our web collection on statistics for biologists contains articles on many of the points above.

Software and code

Policy information about availability of computer code

Data collection	Following Illumina Nextera XT protocol, for each sample 6 Nextera reactions were set up, each with 1 ng of purified ORF DNA. Each reaction was indexed with unique i7/i5 index pairs. After the limited-cycle PCR step, the Nextera reactions were purified with AMPure XP kit. All samples were then pooled and sequenced with Illumina Novaseq S4 platform.
Data analysis	NovaSeq S4 data were processed with software 'AnalyzeSaturationMutagenesis' developed by Broad Institute. Typically, the pair-end reads were aligned to reference sequence. Multiple filters were applied, and some reads were trimmed. The counts of detected variants were then tallied. The output files from AnalyzeSaturationMutagenesis, one for each screening sample, were then parsed, annotated merged into a single .cvs file that is ready for candidate analysis. Software tools are deposited at github.com and available for download.<br>ASM_parser: https://github.com/broadinstitute/SatMut_ASM_Parser/releases/tag/v1.9<br>VariantLibrary_Designer_v1.9: https://github.com/broadinstitute/SatMut_VariantLibrary_Designer/releases/tag/v1.9

For manuscripts utilizing custom algorithms or software that are central to the research but not yet described in published literature, software must be made available to editors and reviewers. We strongly encourage code deposition in a community repository (e.g. GitHub). See the Nature Portfolio guidelines for submitting code & software for further information.

Data

Policy information about availability of data

All manuscripts must include a data availability statement. This statement should provide the following information, where applicable:

- Accession codes, unique identifiers, or web links for publicly available datasets
- A description of any restrictions on data availability
- For clinical datasets or third party data, please ensure that the statement adheres to our policy

All previously unpublished saturation mutagenesis data generated during this study are included in this published article (and its Extended Data information files).

Human research participants

Policy information about studies involving human research participants and Sex and Gender in Research.

Reporting on sex and gender	N/A
Population characteristics	N/A
Recruitment	N/A
Ethics oversight	N/A

Note that full information on the approval of the study protocol must also be provided in the manuscript.

Field-specific reporting

Please select the one below that is the best fit for your research. If you are not sure, read the appropriate sections before making your selection.

[x] Life sciences   [ ] Behavioural & social sciences   [ ] Ecological, evolutionary & environmental sciences

For a reference copy of the document with all sections, see nature.com/documents/nr-reporting-summary-flat.pdf

Life sciences study design

All studies must disclose on these points even when the disclosure is negative.

Sample size	All proliferation and drug treatment assays were performed using at least two or three technical replicates. No sample size calculation was performed for in vitro experiments and the size was chosen based on prior experience.
Data exclusions	No data were excluded.
Replication	All experiments were repeated with at least three biological replicates. All attempts at replication were successful.
Randomization	Not applicable to this study since our experiments did not require this.
Blinding	Investigators were not blinded to sample allocation for any of the experiments since our experiments did not require this.

Reporting for specific materials, systems and methods

We require information from authors about some types of materials, experimental systems and methods used in many studies. Here, indicate whether each material, system or method listed is relevant to your study. If you are not sure if a list item applies to your research, read the appropriate section before selecting a response.

Materials & experimental systems

n/a	Involved in the study
☐	☒ Antibodies
☐	☒ Eukaryotic cell lines
☒	☐ Palaeontology and archaeology
☒	☐ Animals and other organisms
☒	☐ Clinical data
☒	☐ Dual use research of concern

Methods

n/a	Involved in the study
☒	☐ ChIP-seq
☒	☐ Flow cytometry
☒	☐ MRI-based neuroimaging

Antibodies

Antibodies used

Immunoblot analyses were done with phospho-specific antibodies to EGFR (Cell Signaling Technology, catalogue no. 3777, dilution 1:1000), AKT (Cell Signaling Technology, catalogue no. 4060, dilution 1:1000), MEK1/2 (Cell Signaling Technology, catalogue no. 9121, dilution 1:1000), RB1 (Cell Signaling Technology, catalogue no. 8516, dilution 1:1000), ERK1/2 (Cell Signaling Technology, catalogue no. 4370, dilution 1:1000), STAT1 (Cell Signaling Technology, catalogue no. 9167, dilution 1:1000), STAT3 (Cell Signaling Technology, catalogue no. 9145, dilution 1:1000) and with antibodies recognizing total EGFR (Cell Signaling Technology, catalogue no. 2232, dilution 1:1000), AKT (Cell Signaling Technology, catalogue no. 58295, dilution 1:1000) RB (Cell Signaling Technology, catalogue no. 9309, dilution 1:1000) MEK1/2 (Cell Signaling Technology, catalogue no. 4694, dilution 1:500) ERK1/2 (Cell Signaling Technology, catalogue no. 9102, dilution 1:1000) CCNB1 (Cell Signaling Technology, catalogue no. 4138, dilution 1:1000), STAT1 (Cell Signaling Technology, catalogue no. 14994, dilution 1:1000) and STAT3 (Cell Signaling Technology, catalogue no. 9139, dilution 1:1000) to control for total protein expression. Antibody for β-actin (Sigma, catalogue no. AC15, dilution 1:5000) and Vinculin (EMD Millipore, catalogue no. 05-386, dilution 1:10000) were used to verify equivalent loading of total cellular protein. The following secondary antibodies were used Goat anti-Mouse IgG, IgM, IgA (H+L) Secondary Antibody, Alexa Fluor 488 (Life Technologies, catalogue no. A10667, dilution 1:10000) and Donkey anti-Rabbit IgG (H+L) Highly Cross-Adsorbed Secondary Antibody, Alexa Fluor 568 (Life Technologies, catalogue no. A10042, dilution 1:10000).

Validation

Only commercially available antibodies were used in this study. Each has been previously reported in the literature and validated through either over-expression, knockdown, or activity assays by the antibodies manufacturer.

Immunoblot analyses were done with phospho-specific antibodies to:
EGFR (https://www.cellsignal.com/products/primary-antibodies/phospho-egf-receptor-tyr1068-d7a5-xp-rabbit-mab/3777)
AKT (https://www.cellsignal.com/products/primary-antibodies/phospho-akt-ser473-d9e-xp-rabbit-mab/4060)
MEK1/2 (https://www.cellsignal.com/products/primary-antibodies/phospho-mek1-2-ser217-221-antibody/9121)
RB1 (https://www.cellsignal.com/products/primary-antibodies/phospho-rb-ser807-811-d20b12-xp-rabbit-mab/8516)
ERK1/2 (https://www.cellsignal.com/products/primary-antibodies/phospho-p44-42-mapk-erk1-2-thr202-tyr204-d13-14-4e-xp-rabbit-mab/4370)
STAT1 (https://www.cellsignal.com/products/primary-antibodies/phospho-stat1-tyr701-58d6-rabbit-mab/9167)
STAT3 (https://www.cellsignal.com/products/primary-antibodies/phospho-stat3-tyr705-d3a7-xp-rabbit-mab/9145)

Immunoblot analyses were done with specific antibodies to:
EGFR (https://www.cellsignal.com/products/primary-antibodies/egf-receptor-antibody/2232)
AKT (https://www.cellsignal.com/products/primary-antibodies/akt-pan-e7j2c-mouse-mab/58295)
RB (https://www.cellsignal.com/products/primary-antibodies/rb-4h1-mouse-mab/9309)
MEK1/2 (https://www.cellsignal.com/products/primary-antibodies/mek1-2-l38c12-mouse-mab/4694)
ERK1/2 (https://www.cellsignal.com/products/primary-antibodies/p44-42-mapk-erk1-2-antibody/9102)
CCNB1 (https://www.cellsignal.com/products/primary-antibodies/cyclin-b1-antibody/4138)
STAT1 (https://www.cellsignal.com/products/primary-antibodies/stat1-d1k9y-rabbit-mab/14994)
STAT3 (Cell Signaling Technology, catalogue no. 9139, dilution 1:1000)
β-actin (https://www.thermofisher.com/antibody/product/beta-Actin-Antibody-clone-AC-15-Monoclonal/MA1-91399)
Vinculin (https://www.emdmillipore.com/US/en/product/Anti-Vinculin-Antibody-clone-V284,MM_NF-05-386)
Goat anti-Mouse IgG, IgM, IgA (H+L) Secondary Antibody, Alexa Fluor 488 (https://www.thermofisher.com/antibody/product/Goat-anti-Mouse-IgG-H-L-Cross-Adsorbed-Secondary-Antibody-Polyclonal/A-11001)
Donkey anti-Rabbit IgG (H+L) Highly Cross-Adsorbed Secondary Antibody, Alexa Fluor 568 (https://www.thermofisher.com/antibody/product/Donkey-anti-Rabbit-IgG-H-L-Highly-Cross-Adsorbed-Secondary-Antibody-Polyclonal/A10042?gclid=EAIaIQobChMI5OKHuLqmggMVTBCtBh3urgo2EAAYASAAEgL1XvD_BwE&ef_id=EAIaIQobChMI5OKHuLqmggMVTBCtBh3urgo2EAAYASAAEgL1XvD_BwE:G:s&s_kwcid=AL!3652!3!605801922206!p!!g!!donkey%20anti%20rabbit%20568!596889499!110491006544&cid=bid_pca_aus_r01_co_cp1359_pjt0000_bid00000_0se_gaw_nt_pur_con)

Eukaryotic cell lines

Policy information about cell lines and Sex and Gender in Research

Cell line source(s)

The HCC4006 (catalogue no. CRL-2871) lung cancer cell line was purchased from ATCC, and PC9 and HCC827 lung cancer cell lines were obtained from the Cancer Cell Line Encyclopedia (CCLE; RRID:SCR_013836) and DepMap (Cancer Dependency Map Portal, RRID:SCR_017655). The sources for these lung cancer lines are listed at DepMap.org, and they can be obtained from their respective sources. Identities were confirmed by single-nucleotide polymorphism array.

Authentication	Cell line identities were reconfirmed by short tandem repeat (STR) profiling at Dana-Farber Cancer Institute (Molecular Diagnostics Laboratory Core).
Mycoplasma contamination	Cell lines were confirmed negative for Mycoplasma infection (MycoStrip™ - Mycoplasma Detection Kit)
Commonly misidentified lines (See ICLAC register)	No commonly misidentified cell lines were used in this study.
