# Supplementary material for: Comprehensive mutational scanning of EGFR reveals TKI sensitivities of extracellular domain mutants
Source: Nat Commun. 2024 Mar 28;15:2742. doi: 10.1038/s41467-024-45594-4 (PMC10978866; doi:10.1038/s41467-024-45594-4)
Supplement: Supplementary file 3 — Description of Additional Supplementary Files [file 41467_2024_45594_MOESM3_ESM.pdf]

Supplementary Data 1. Functional EGFR variant enrichment scores. Data are presented as Z-score values. A two-tailed t-test was performed to calculate p-values for each EGFR variant.

Supplementary Data 2. Identification of novel EGFR variants. Data are presented as Z-score values, with a cut-off of Z-score > 2. A two-tailed t-test was performed to calculate p-values for each EGFR variant. A literature comparison was performed to determine EGFR variant overlap from other EGFR variant studies.
