# Supplementary material for: Comprehensive mutational scanning of EGFR reveals TKI sensitivities of extracellular domain mutants
Source: Nat Commun. 2024 Mar 28;15:2742. doi: 10.1038/s41467-024-45594-4 (PMC10978866; doi:10.1038/s41467-024-45594-4)
Supplement: Supplementary file 7 — Source Data [file 41467_2024_45594_MOESM7_ESM.zip › Hayes Source Data Legends.docx]

**Source information**

**Figures 1b, 3b, 3c, 4b, 4h, 5a, 5b, 5c, 5d, 6b, 6c, 7a**

**Supplemental Figures 1a, 4b, 6a, 6b, 7a**

Dose response data displayed as normalization of cell titer glo luminescence scores. Please check corresponding figure legends and methods for more information.

Calculations were made as follows:

- Subtract background from individual wells
- Calculate average of vehicle wells (3x)
- Divide each single well by vehicle average
- Average technical replicates (3x)
- Multiple by 100
- Plot data points in Prism

**Figures 1e, 1g, 3i, 3l, 4g, 7d**

**Supplemental Figures 1d, 1e, 5e, 5f, 5i, 5j, 6e, 6f, 6d, 6e, 6f, 7h**

Signal transduction data displayed as normalization of phospho- to total protein ratio as measured by densitometry scores. Please check corresponding figure legends and methods for more information.

Calculations were made as follows:

- Calculate MOCK phospho-protein/total-protein ratio after vehicle treatment
- For remaining conditions +/- inhibitor calculate phospho-protein/total-protein ratio
- Divide all conditions by MOCK phospho-protein/total-protein ratio after vehicle treatment
- Plot data points in Prism
